# Investigating the interaction between schizotypy, divergent thinking and cannabis use

**DOI:** 10.1016/j.concog.2011.11.009

**Published:** 2012-03

**Authors:** Gráinne Schafer, Amanda Feilding, Celia J.A. Morgan, Maria Agathangelou, Tom P. Freeman, H. Valerie Curran

**Affiliations:** a Clinical Psychopharmacology Unit, Research Department of Clinical, Health and Educational Psychology, University College London, Gower Street, London WC1E 6BT, United Kingdom; b The Beckley Foundation, Beckley Park, Oxford OX3 9SY, United Kingdom

**Keywords:** Cannabis, Schizotypy, Creativity

## Abstract

Cannabis acutely increases schizotypy and chronic use is associated with elevated rates of psychosis. Creative individuals have higher levels of schizotypy, however links between cannabis use, schizotypy and creativity have not been investigated. We investigated the effects of cannabis smoked naturalistically on schizotypy and divergent thinking, a measure of creativity. One hundred and sixty cannabis users were tested on 1 day when sober and another day when intoxicated with cannabis. State and trait measures of both schizotypy and creativity were administered. Quartile splits compared those lowest (*n* = 47) and highest (*n* = 43) in trait creativity. Cannabis increased verbal fluency in low creatives to the same level as that of high creatives. Cannabis increased state psychosis-like symptoms in both groups and the high creativity group were significantly higher in trait schizotypy, but this does not appear to be linked to the verbal fluency change. Acute cannabis use increases divergent thinking as indexed by verbal fluency in low creatives.

## Highlights

► We study the acute effects of cannabis on divergent thinking and schizotypy. ► Quartile splits compared those lowest and highest in trait creativity. ► Those higher in trait creativity also had higher trait schizotypy. ► State schizotypy increased for both groups when intoxicated with cannabis. ► Acutely, cannabis increases verbal fluency in individuals low in trait creativity.

## 1 Introduction

Cannabis is the world’s most popular illicit drug with the United Nations estimating 190 million users each year (UNODC, 2010). Cannabis has received considerable research and media attention due to evidence of its link with both psychosis and dependence. Alongside these negative effects, it has been suggested both anecdotally and in the scientific literature that cannabis may improve creativity. A recent review concluded that over 50% of cannabis users self report that enhanced creativity results from their acute cannabis use (Green, Kavanagh, & Young, 2003). The link between creativity and cannabis use is currently a topic of lively debate given suggestions that ‘writer’s block’ is one of a variety of reasons for obtaining a prescription of ‘medical marijuana’ in some North America States (St. Pierre, as quoted in Parloff (2009)).

However, the link between cannabis and creativity has not been extensively studied, partly due to a lack of consensus on exactly what creativity is or how it can be objectively measured. One model of creative thinking was developed by Guilford (1967), who conceptualised creativity as a distinction between divergent and convergent thinking. Divergent thinking refers to the fluency and flexibility of new representations in working memory. It is used to generate answers to open-ended questions or problems and often yields novel ideas and solutions (Reber, 1995). Fluency tasks have been suggested as appropriate measures of divergent thinking (Guilford, 1959). In contrast, convergent thinking is often linked with concrete problem-solving through pooling knowledge or information to converge on a single correct solution (Reber, 1995). More recently, it has been suggested that convergent thinking is also important in creative problem solving as it allows a person to be able to select out the good ideas in their results from divergent thinking (Csikszentmihalyi, 1996). The Remote Associates Test (RAT; Mednick, 1962) is used as a measure of convergent thinking as it requires participants to converge on a single target word to connect three semantically unrelated words. It may be used as a general measure of creativity as it also requires processes similar to divergent thinking to generate many remote associates before converging on the correct answer (Snyder & Lopez, 1995).

A few studies have attempted to objectively investigate whether cannabis increases creativity. Cannabis users produced more original (i.e. statistically infrequent) responses on an associative ability test when intoxicated with the drug compared to users given placebo (Block, Farinpour, & Braverman, 1992). Similarly, verbal fluency was significantly and dose-dependently improved by THC (the active ingredient in cannabis) compared with placebo in healthy volunteers given all treatments (Curran, Brignell, Fletcher, Middleton, & Henry, 2002). In contrast, Tinklenberg, Darley, Roth, Pferbhaum, and Koppel (1978) found that cannabis did not enhance scores in uniqueness, fluency, flexibility or elaboration during an object description task, considered to be a measure of creativity.

Greater self-reported frequency of cannabis use has been found to be associated with higher creativity scores, as measured by the Personal Opinion Survey, a questionnaire used to measure creativity (Eisenman, Grossman, & Goldstein, 1980).

Though it is not yet known exactly how cannabis use might contribute to an increase in creativity, it is possible that schizotypy may play a mediating role. Schizotypy describes a continuum in the general population of cognitive characteristics and experiences ranging from mild dissociative states to more extreme states linked to psychosis and schizophrenia. It has been linked to creativity in many studies (e.g. Nettle, 2006; Rawlings & Locarnini, 2008). Higher levels of schizotypy have also been found across populations of cannabis users compared to ex-users and drug-free controls (Skosnik, Spatz-Glenn, & Park 2001; Williams, Wellman, & Rawlins, 1996). Further, Mason et al. (2009) found that smoking cannabis in a naturalistic setting induced significant increases in psychotomimetic symptoms across all users, and that this was particularly marked in individuals with higher trait schizotypy. A recent study also found that naturalistically smoked cannabis enhanced semantic priming, leading to quicker response times to distant concepts (Morgan, Rothwell, Mason, & Curran, 2010). This increased activation of concepts and ideas is similar to Guilford’s (1967) conception of divergent thinking and also is one of the key processes Mednick (1962) suggested to be involved in creative thinking. Therefore by a similar process it is possible that cannabis may enhance creative thinking.

The aim of the current study was to examine the effects of cannabis use on the interplaying variables of state schizotypy and state creativity by comparing cannabis users high and low in trait creativity. It set out to investigate state creativity using two tasks assessing convergent and divergent thinking respectively: the Remote Associates Task (RAT: Mednick, 1962) and verbal fluency. We predicted that acutely cannabis would increase state schizotypy, in line with previous studies (e.g. Mason et al., 2009), and increase state creativity (Block et al., 1992; Curran et al., 2002).

## 2 Methods

### 2.1 Participants and design

One hundred and sixty cannabis users were recruited using snowball sampling (Solowij, Hall, & Lee, 1992). On 1 day participants smoked their own cannabis, on another (non-intoxicated) day, either 7 days later or 7 days before, they were drug-free (verified for drugs other than cannabis by urine analysis). Participants’ state (i.e. intoxicated or non-intoxicated) was counterbalanced across the 2 days. An independent groups, repeated measures design was used to compare the cannabis users highest and lowest in trait creativity on the two separate test days. The study was approved by the UCL Graduate School Research Ethics Committee.

### 2.2 Procedure

Both test sessions were conducted in a naturalistic setting – a quiet room that they would normally smoke in. Participants provided written informed consent to participate in this study on both test days. For the non-intoxicated test session, participants were required to be abstinent from cannabis for a minimum of 24 h and this was verified by analysis of saliva samples. During the intoxicated session, participants were assessed after smoking a ‘spliff’ of their own cannabis. A sample of their cannabis was taken to be analysed for levels of THC. Participants were reimbursed for their time. The assessments reported below were administered in the same order on both test days and formed part of a broader test battery the results of which are described elsewhere (Morgan, Freeman, et al., 2010; Morgan, Rothwell, et al., 2010; Morgan, Schafer, et al., 2010).

### 2.3 Assessment

#### 2.3.1 Both test days

*Psychotomimetic States Inventory (PSI*; Mason et al., 2009): This 48 item questionnaire assesses state psychotomimetic symptoms and yields a total score and sub-scales of: ‘Delusory Thinking’, ‘Perceptual Distortions’, ‘Cognitive Disorganization’, ‘Anhedonia’, ‘Mania’ and ‘Paranoia’. Each item is rated on a 4-point scale.
*Verbal fluency task*: A divergent thinking task used here to assess state creativity. Participants were required to give as many verbal responses linked to a letter of the alphabet, by saying as many words in 60 s as they could think of that begin with the given letter (either a ‘B’, or ‘M’). Data were scored in total number correct, perseverative errors and other errors.
*Category fluency task*: This task tests semantic fluency. Participants were required to give as many verbal responses linked to a named category (either four legged animals or fruit) in 60 s. Data were scored in the same way as the verbal fluency task.
*The Remote Associates Test (RAT;* Mednick, 1958): This task tapped creative thinking involving both convergent and divergent thinking. It consisted of 16 word triads (e.g. *night*, *wrist*, and *stop*) which participants were instructed to generate a word related to all the three words in the set (e.g. *watch*). Participants were given 4 min to find words that fitted in each triad. This requires both divergent and convergent thinking as participants must go through the process of generating many (remote) associates before inhibiting incorrect answers (Snyder & Lopez, 1995). The outcome variable was total number of problems solved.

#### 2.3.2 Additional measures on non-intoxicated day only

*Schizotypal Personality Questionnaire (SPQ*; Raine, 1991): A self-report scale assessing trait schizotypy. There were 74 questions to which a participant must give a “Yes/No” response. Scores yielded three factors of schizotypy: (Cognitive-Perceptual, Interpersonal, Disorganised) and a total score.
*Creative Achievement Questionnaire (CAQ*; Carson, Peterson, & Higgins, 2005): This is a self-assessment of participants’ recognised achievements within various creative domains, as was used as a measure of trait creativity: It assesses creative achievements in the following domains: visual arts; music; dance; architectural design; entrepreneurial ventures; creative writing; humour; inventions; scientific enquiry; theatre and films; culinary arts. It is composed of three parts: self-perceived talent, concrete achievements and peer-perceived talent. The CAQ total is calculated by summing the concrete achievements.
*Weschler Test of Adult Reading (WTAR*; Wechsler, 2001): This was used as an assessment of premorbid verbal IQ.
*Spielberger Trait Anxiety Inventory (STAI*; Spielberger, 1983): The STAI comprises of twenty questions designed to measure trait anxiety levels.
*Severity of Dependence Scale (SDS*; Gossop et al., 1995): A short five-item questionnaire regarding drug use, this was used to measure cannabis dependence.
*Beck Depression Inventory* (*BDI*; Beck, Ward, & Mendelson, 1961): The BDI measures the severity of depression. Responses range from 0 to 3, to indicate the severity of each symptom.

### 2.4 Statistical analysis

Data were analysed using SPSS version 16 (Chicago, IL). In order to assess cannabis users high and low in creativity, 90 participants were selected from the top and bottom quartiles of CAQ totals. Participants in the low creative group (*n* = 43) had a score of 4 or lower, participants in the high creative group (*n* = 47) had a CAQ total of 12 and above. For demographic and trait data, *t*-tests and chi-squared tests were used as appropriate. State schizotypy and creativity data were analysed with repeated measures ANOVAs, with Group (low creativity, high creativity) as a between and Day (intoxicated, non-intoxicated) as a within subjects factor. Interactions were explored with a maximum of four post-hoc *t*-tests, Bonferroni corrections reduced the significance level to 0.0125.

## 3 Results

### 3.1 Demographics (Table 1)

There were no group differences in gender, years in education, premorbid IQ (WTAR) or days of cannabis use per month. A significant group difference was found in age [*t*(88) = −3.27, *p *= .002)] and schizotypy [*t*(82) = −3.48, *p *< .001)]. The high creativity group was over a year older and had higher SPQ scores than the low creativity group. Additionally, there were no differences between groups in other aspects of psychological wellbeing or drug use.

#### 3.1.1 Verbal fluency (divergent thinking)

**Fig. 1:**
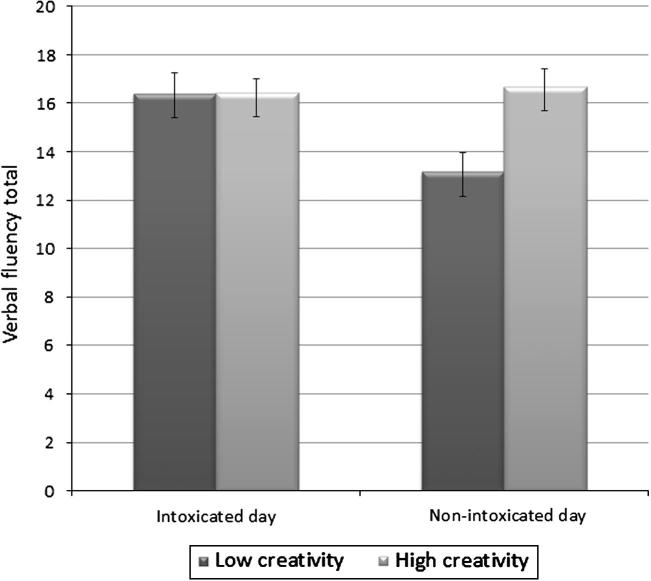
Verbal fluency scores across days in the low and high creativity quartiles. Bars represent ±standard errors.

A 2 × 2 repeated measures ANOVA yielded a significant Day × Group interaction [*F*(1, 85) = 10.39, *p *= .002; Fig. 1] and a main effect of Day [*F*(1, 85) = 9.82, *p *= .002]. Post-hoc *t*-tests showed that the high creativity group did not differ across days but the low creativity group showed a significant improvement in scores on the intoxicated day compared to the non-intoxicated day [*t*(40) = 4.742, *p *< .001]. On the non-intoxicated day, the high creativity group performed significantly better than the low creativity group (*t*(88) = −3.49; *p* = .002).

#### 3.1.2 Category fluency

**Fig. 2:**
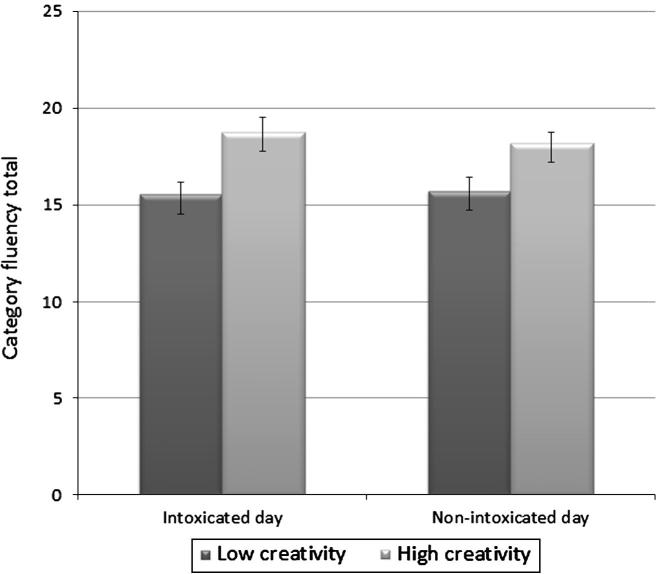
Category fluency scores across days in the low and high creativity quartiles.

A 2 × 2 repeated measures ANOVA yielded a significant main effect of Group [*F*(1, 85) = 10.367, *p *= .002] but no Day × Group interaction or main effect of Day. The high creativity group performed better than the low creativity group on both the intoxicated and non-intoxicated days (Fig. 2).

#### 3.1.3 Creative problem solving (RAT)

A 2 × 2 repeated measures ANOVA revealed trends towards a Day × Group interaction [*F*(1, 85) = 3,60, *p *= .06; Table 2] and towards a main effect of Group [*F*(1, 85) = 3.685, *p *= .06]. Bonferroni corrected post-hoc comparisons showed only a trend for a decreased performance in the RAT in the high creativity group on the intoxicated day [*t*(45) = 2.098, *p *= .042]; the low creativity group scores remained similar across both days (Table 2).

#### 3.1.4 State schizotypy (PSI)

**Fig. 3:**
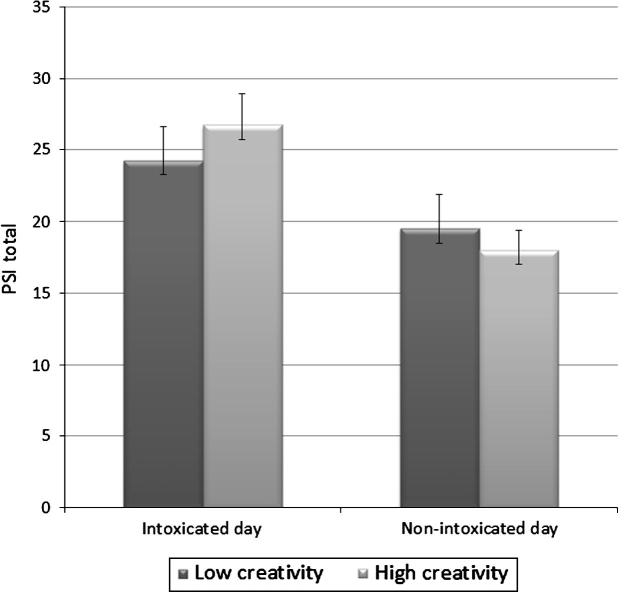
PSI scores across days in the low and high creativity quartiles.

Analysis of PSI data found a main effect of Day [*F*(1, 80) = 14.84, *p *< .001] with both groups having significantly greater PSI scores on the intoxicated day compared to the non-intoxicated day (Fig. 3).

#### 3.1.5 Correlations

In the low creativity group, there was no significant correlation between change in schizotypy (PSI) scores and change in verbal fluency across days.

## 4 Discussion

The main finding of this study was that divergent thinking as indexed by the verbal fluency task was enhanced acutely by cannabis in individuals who were low in trait creativity. Their performance after smoking cannabis improved to the same level as the high creativity group. When not intoxicated, the low creativity group’s performance was significantly worse than the high creativity group. On the category fluency task, the high creativity group significantly outperformed the low creativity group on both testing days so performance was unaffected by cannabis use. The high creativity group had significantly higher SPQ scores than the low creativity group. State schizotypy, as measured by PSI scores, increased significantly for both groups on the intoxicated day, replicating Mason et al. (2009).

The increase in verbal fluency for those low in trait creativity on the intoxicated day supports the notion that cannabis can enhance aspects of creativity (Eisenman et al., 1980). Our findings in a naturalistic study replicate those of a controlled laboratory study in which oral doses (7.5, 15 mg) of THC or matched placebo were administered using double-blind procedures (Curran et al., 2002). It is possible that the high creativity group was performing at ceiling levels on both test days and thus could not increase scores when intoxicated. This possibility is less likely given that the increase in fluency after THC observed by Curran et al. was in the same participants tested over three separate days after low dose, high-dose THC and placebo. Curran et al. suggested that the enhanced verbal fluency following THC reflected some sort of disinhibition of frontal cortical functions which is consistent with cannabis’ known neurobiological effects. This disinhibition may already be present in the high creativity group when not intoxicated and they may already be reaching maximum performance levels that do not change under the influence of cannabis. In the neurobiological model of creativity (Flaherty, 2005), enhanced frontal functioning is thought to play a key role. The observed increase in verbal fluency may be related to the finding that the right hemisphere appears to be involved in recognising remote associates (Rdel, Cook, Regard, & Landis, 1992), and that cannabis acutely enhances right hemispheric activity (Mathew, Wilson, & Turkington, 2002).

In contrast to the verbal fluency, categorical fluency scores on each day did not change significantly within groups. On both test days, the high creativity group scored higher than the low creativity group on category fluency. This difference may be due to the different aspects of divergent thinking the two tasks are tapping. While verbal fluency relies on the phonological cues for word retrieval, categorical fluency relies on semantic associates for retrieval. It has been shown that these two tasks are mediated by different areas of the brain, with verbal fluency linked to the frontal cortex and categorical fluency linked to the temporal cortex (Baldo, Schwartz, Wilkins, & Dronkers, 2006). The high creativity group may have enhanced functioning in the temporal cortex in comparison to the low creativity group and this may not be affected by cannabis use. Acutely, cannabis may also stimulate dopamine release in the mesolimbic pathway which includes the frontal cortex, possibly related to the increase in verbal fluency (Caspi, Moffitt, & Cannon, 2005).

The finding of a significant trait schizotypy difference between groups is in line with Rawlings and Locarnini’s (2008) data strongly linking creativity with schizotypy. State schizotypy (PSI) scores increased significantly on the intoxicated day for both groups, replicating previous findings (Mason et al., 2009). Over days, the increase in PSI in the low creativity group does not appear to be related to their increased verbal fluency as there was no significant correlation between changes in these scores.

The results of the analyses on RAT scores found a trend for a between groups difference, with the high creativity group performing better than the low creativity group. A trend for a decrease in performance following acute cannabis in the high creativity group was also found. While it does not appear that divergent thinking, as measured by verbal fluency is hindered by cannabis use, it may be possible that convergent thinking is affected, leading to the slightly decreased RAT scores when intoxicated. This may have important implications for the possible relationship between cannabis use and creative thinking as whilst divergent thinking encompasses the essence of creativity by allowing for loose associations to be formed between concepts (Reber, 1995), minimal convergent thinking or creative problem solving may hinder the ability to form connections between these ideas. It appears that the level of convergent thinking may largely determine the degree to which one’s thinking is considered either creative (i.e. increased convergent thinking) or psychotic (i.e. decreased convergent thinking), as well as the associated productivity. It is possible the increase in divergent thinking found in the low creativity group may lead to enhanced creative thought, but may not lead to convergence on a specific creative idea. As well as this, there is strong evidence that cannabis impairs episodic memory (e.g. Curran et al., 2002) which may prevent a person from remembering any creative ideas they may have had whilst under the influence of cannabis.

Despite the benefits of a more ecologically valid experiment through cannabis being used in naturalistic settings, issues regarding reliability must be addressed. As creativity is ill-defined and thus has a large subjective component, the verbal fluency task and RAT were used as comparatively easy-to-score measures compared to others used in the literature, in order to ensure high inter-rater reliability. In a number of definitions of creativity, originality appears to be a key inclusive feature. The RAT does not allow for original responses, as there is only one target answer for each of the three-word sets. Whilst the verbal fluency task allows participants more freedom to give a range of responses, the measure is still only based on the quantity of responses. However, Gilhooly, Fioratou, Anthony, and Wynn (2007) found that verbal fluency is related to the production of the number of ‘new’ uses participants came up with on an alternative uses task, with a positive correlation between verbal fluency scores and the number of new uses participants can think of. They hypothesised that this may be due to divergent thinking involving the employment of executive processes, as happens in verbal fluency. In a future related study to support that the tasks did in fact tap into aspects of creativity – not unconnected aspects of cognitive performance – a task involving a measure of originality would be fundamental. Block et al.’s (1992) methodology of analysing original responses by looking at those considered statistically infrequent may be an appropriate alternative.

Another limitation relates to the reductionist approach of measuring creativity using word-based tasks alone. This fails to encompass the multi-faceted nature of creativity as outlined by Guilford (1959). This is highlighted when comparing the currently used state creativity measures to the various aspects of creativity measured in the CAQ. Nevertheless, issues regarding the CAQ stem from its focus on nationally recognised works of creative achievement. Given that the current sample was aged between 18 and 24, a relatively young test group, it may be unrealistic for any potential works of artistic merit to have achieved public recognition; the use of recognised achievements, talents or prizes as a way of scoring creative achievements may not pick up on individuals who may still be creative but may not have received the appropriate recognition to obtain a high CAQ score. Therefore the validity of using the CAQ as a measure of innate creativity for younger individuals may be questionable. However, this has not prevented other studies from using the CAQ on similar populations. Carson, Peterson, and Higgins (2003) used a group with a mean age of 20.7 years in their study on creativity, intelligence and latent inhibition. In the study, they compared *eminent creative achievers* (those under the age of 21 who reported unusually high scores in specific domains of creative achievement) with those low in creative achievement. While we only looked at the top and bottom quartiles and not at *eminent creative achievers*, it may be possible higher creativity scores at a young age may still be indicative of trait creativity.

## 5 Conclusion

Cannabis use may increase an aspect of creativity (verbal generation) in cannabis users with low trait creativity, though convergent thinking may be required in order to generate more meaningful associations from divergent thinking. While the high creativity group had significantly higher scores in trait schizotypy, the increased divergent thinking in the low creativity group does not appear to be linked to the higher states of psychosis also induced by the effects of cannabis. The study provides possible indirect support for the theory that divergent thinking is related to a disinhibition of frontal cortical functions.

## Figures and Tables

**Table 1:** Means (Standard Deviations) for demographic, questionnaire totals, THC data and verbal fluency scores across cannabis users split between different quartiles in trait creativity.

	Low creativity (*n *= 43)	2nd Quartile (*n *= 35)	3rd Quartile (*n *= 32)	High creativity (*n *= 47)
Age⁎	20.37 (2.02)	20.03 (2.43)	20.57 (2.36)	21.62 (1.58)
Years in education	14.46 (1.86)	14.14 (2.44)	14.2 (2.33)	14.72 (2.23)
WTAR	40.77 (7.87)	41.74 (7.23)	44.53 (4.38)	43.15 (5.27)
Gender (no. females/males)	10/33	9/26	10/22	16/31
Schizotypal personality questionnaire⁎	11.33 (9.34)	18.94 (12.4)	16.53 (11.9)	18.64 (9.94)
Creative achievements questionnaire⁎⁎	2.16 (1.43)	6.17 (0.82)	9.5 (1.14)	17.32 (6.51)
Spielberger trait anxiety inventory	40.15 (10.01)	40.03 (8.66)	39.66 (9.01)	39.50 (9.39)
Beck depression inventory	7.41 (7.41)	7.62 (7.04)	7.87 (7.21)	7.76 (8.29)
Severity of dependence scale	3.14 (3.59)	2.43 (2.54)	2.16 (2.86)	3.51 (3.21)
Days cannabis used per month	14.95 (11.51)	15.90 (10.80)	14.50 (10.10)	17.09 (10.81)
Age of first regular cannabis use	16.99 (2.62)	15.80 (2.36)	15.90 (2.04)	16.51 (2.05)
% THC in cannabis sample	9.30 (4.54)	11.00 (4.22)	9.61 (3.9)	10.50 (5.01)
Days alcohol drunk per month	8.25 (6.52)	11.6 (8.26)	12.7 (6.1)	11.26 (8.31)
Salivary THC intox day (ng/mL)	25.65 (59.76)	27.50 (60.10)	17.70 (27.00)	34.62 (52.86)
Salivary THC non-intox day (ng/mL)	3.42 (9.97)	1.00 (2.48)	1.06 (2.32)	1.54 (6.63)
Intoxicated Verbal fluency score	16.39 (5.43)	15.15 (6.33)	15.60 (5.18)	16.43 (3.96)
Non-intoxicated verbal fluency score⁎⁎	13.32 (5.27)	14.42 (4.93)	15.90 (3.88)	16.55 (5.17)

THC, Δ9-tetrahydrocannabidiol.

⁎ *p* < .01.

⁎⁎ *p* < .001.

**Table 2:** Means (Standard Deviations) of RAT scores for creativity quartiles on non-intoxicated and intoxicated days.

	Low creativity	2nd Quartile	3rd Quartile	High creativity
Intoxicated day	4.71 (3.03)	5.09 (2.70)	5.32 (2.39)	4.98 (2.57)
Non-intoxicated day	4.53 (2.53)	4.94 (3.03)	5.64 (2.48)	6.04 (2.81)
